# Isolated Anti‐SS‐A Antibody Seropositivity as a Poor Prognostic Factor in Systemic Sclerosis: Insights From a Cohort of 307 Cases

**DOI:** 10.1111/1346-8138.70055

**Published:** 2025-11-22

**Authors:** Nana Ishida, Kazuki M. Matsuda, Hirohito Kotani, Hayakazu Sumida, Shinichi Sato

**Affiliations:** ^1^ Department of Dermatology The University of Tokyo Graduate School of Medicine Tokyo Japan; ^2^ Scleroderma Center, The University of Tokyo Hospital Tokyo Japan; ^3^ SLE Center The University of Tokyo Hospital Tokyo Japan

**Keywords:** autoantibody, Cox regression analysis, interstitial lung disease, SSA, systemic sclerosis

## Abstract

Systemic sclerosis is an autoimmune disease characterized by vasculopathy, fibrosis, and immune dysregulation. Anti‐SS‐A antibodies (anti‐SSA) have been reported to confer severe clinical manifestations in some Western and Japanese cohorts. We aimed to determine whether anti‐SSA seropositivity affects clinical outcomes in Japanese patients. We retrospectively analyzed 307 Japanese patients with systemic sclerosis who underwent initial evaluation between January 2011 and March 2020 in our clinic. “Isolated” anti‐SSA seropositivity was defined as positivity for anti‐SSA in the absence of anti‐topoisomerase I, anti‐centromere, anti‐RNA polymerase III, and anti‐U1 ribonucleoprotein antibodies. Overall survival was defined as time to all‐cause mortality, and progression‐free survival was defined as time to disease progression necessitating intensified therapy. Cox proportional hazards models were employed to estimate hazard ratios and 95% confidence intervals. For patients with “isolated” anti‐SSA seropositivity, further investigation for SSc‐related autoantibodies was conducted utilizing Autoantibody Array Assay (A‐Cube). Anti‐SSA were detected in 31.3% of patients. Although anti‐SSA seropositivity overall correlated with interstitial lung disease, it was not independently associated with overall survival or progression‐free survival. In contrast, “isolated” anti‐SSA seropositivity emerged as an independent risk factor for both shorter overall survival (hazard ratio 21.7, 95% confidence interval 5.57–84.8) and progression‐free survival (hazard ratio 4.18, 95% confidence interval 1.05–16.7). Expanded serologic testing revealed additional autoantibodies implicated in severe SSc phenotypes. These findings underscore the importance of routinely assessing anti‐SSA and highlight the need for autoantibody screening in depth in this subpopulation.

## 1 Introduction

Systemic sclerosis (SSc) is characterized by a triad of aberrant immune activation, vasculopathy, and progressive fibrosis, which together culminate in significant morbidity and mortality [1]. The classical pathogenesis model involves widespread microvascular endothelial injury, dysregulated inflammation, and excessive collagen deposition by activated myofibroblasts [2, 3]. Over time, these processes lead to end‐organ damage involving the skin, lungs, kidneys, and heart [4]. Autoantibodies are nearly universal in SSc (detected in > 90% of patients) and not only serve as valuable diagnostic markers but also correlate with distinct disease phenotypes and outcomes [5]. Among the most frequently studied are anti‐topoisomerase I (ATA), anti‐centromere (ACA), anti‐RNA polymerase III (ARA), and anti‐U1 ribonucleoprotein (RNP) antibodies (Abs), each of which defines clinical subsets with characteristic organ complications and prognostic implications [6].

Recent attention has focused on the clinical significance of anti‐SS‐A (Ro52/60) Abs (anti‐SSA) in SSc. Although anti‐SSA is classically associated with Sjögren's syndrome (SS) and systemic lupus erythematosus (SLE), multiple cohort studies have detected anti‐SSA in 15%–20% (or more) of SSc patients [7]. Emerging evidence suggests that anti‐SSA seropositivity in SSc is linked to an increased risk of severe organ involvement. For instance, analyses from German and Canadian SSc registries have indicated that anti‐SSA seropositivity confers approximately 2–3‐fold higher odds of developing interstitial lung disease (ILD) [8, 9]. A Japanese cohort study has similarly reported that anti‐SSA seropositivity may be an independent risk factor for ILD and more aggressive skin disease [10]. The study also revealed that patients positive for anti‐SSA alone had a greater susceptibility to ILD than those positive for both anti‐SSA and SSc‐specific autoantibodies. Nonetheless, the impact of anti‐SSA on overall survival (OS) and progression‐free survival (PFS) in SSc remains unclear, partly due to the lack of longitudinal observation and potential overlap with other disease‐specific Abs. Moreover, whether patients who have “isolated” anti‐SSA seropositivity (i.e., lacking other major SSc‐related Abs) exhibit distinct clinical phenotypes or outcomes compared to those with coexisting autoantibody profiles has not been fully elucidated.

In this context, we conducted a retrospective cohort study of Japanese SSc patients to investigate the clinical significance of anti‐SSA seropositivity, with particular focus on whether isolated anti‐SSA status might be associated with unique disease manifestations or outcomes, including OS and PFS. Here, we report that approximately one‐third of our study cohort was anti‐SSA‐positive, and that isolated anti‐SSA seropositivity was significantly linked to adverse survival outcomes and disease progression, independent of established risk factors. These findings support the inclusion of anti‐SSA in routine serologic assessment and underscore the potential utility of “isolated” anti‐SSA seropositivity as a marker of higher‐risk SSc.

## 2 Methods

### 2.1 Patient Enrollment and Autoantibody Profiling

We recruited Japanese SSc patients who initially arrived at our clinic from April 2011 until March 2020, all of whom fulfilled the classification criteria established by the American College of Rheumatology and European League Against Rheumatism in 2013 [11]. Serum levels of ATA, ACA, and ARA were examined by enzyme‐linked immunosorbent assays. Serum anti‐U1‐RNP Ab, anti‐Sm Ab, anti‐SSA, and anti‐SS‐B Ab (anti‐SSB) positivity were explored by chemiluminescent enzyme immunoassays. Isolated anti‐SSA seropositivity was defined as the presence of anti‐SSA without detectable levels through clinical laboratory testing covered by health insurance in Japan (ATA, ACA, ARA, and anti‐U1‐RNP Abs). In individuals with isolated anti‐SSA seropositivity, further autoantibody investigations were conducted using indirect immunofluorescent assays with Hep‐2 cells and Autoantibody Array Assay (A‐Cube; Fushimi Pharmaceutical Co. Ltd., Kagawa, Japan), a multiplex assay with a high concordance with immunoprecipitation [12], to assess additional serological features. A‐Cube included 13 SSc‐associated autoantibodies, 2 associated with primary biliary cholangitis (PBC), 17 associated with dermatomyositis (DM) or polymyositis (PM), and 11 associated with overlap syndromes such as SLE or SS. The whole study was approved by the ethical committee of The University of Tokyo Hospital (approval number: 0695 and 2023051G), and the study complied with the Declaration of Helsinki guidelines. Written informed consent was obtained from the participants.

### 2.2 Clinical Assessment

Clinical data of the patients were gathered by retrospective review of electronic medical records. Demographic information, laboratory results, examination findings, and concomitant medications were obtained at the time closest to the first arrival to our clinic. Onset of the disease was defined as the first clinical event that was a clear manifestation of SSc other than Raynaud's phenomenon. Disease duration was measured from the first non‐Raynaud's phenomenon manifestation to the initial visit to our clinic. Patients were categorized by LeRoy's classification rule into diffuse cutaneous SSc (dcSSc) or limited cutaneous SSc [13]. Skin thickness was semi‐quantitatively examined by the modified Rodnan total skin thickness score (mRSS) [14]. Presence of organ involvement was determined as previously described [15]. Briefly, ILD was defined as bibasilar interstitial fibrosis on high‐resolution computed tomography. Pulmonary hypertension was defined as mean pulmonary artery pressure greater than 25 mmHg on right heart catheterization. Scleroderma renal crisis (SRC) was framed as malignant hypertension and/or rapidly progressive renal dysfunction. Reflux esophagitis was defined as Grade M or more on Los Angeles classification on the basis of gastrointestinal endoscopy [16]. The presence of ileus was determined upon the clinician's diagnosis. Concurrence of SLE, SS, PM/DM, PBC, and anti‐phospholipid Ab syndrome (APS) was determined in compliance with their classification criteria previously established [17, 18, 19, 20, 21]. OS was defined as the duration from the patient's first visit to our institution until death from any cause. PFS was defined as the time from the first visit to the point of treatment modification due to any exacerbation of SSc. Concomitant immunosuppressants included cyclophosphamide, mycophenolate mofetil, tacrolimus, cyclosporine, azathioprine, and mizoribine.

### 2.3 Statistical Analysis

Two group comparisons were performed by two‐sided Mann–Whitney *U*‐test for continuous variables, and two‐sided Fisher's exact test for categorical variables. Both univariate and multivariate survival time analyses were conducted by Cox regression analysis and visualized by the Kaplan–Meier method. Analyses were performed using Stata/IC 15 (StataCorp LLC, TX, USA), R, RStudio, and R packages “dplyr,” “UpSetR,” and “corrplot”. We set the threshold for statistical significance at *p* < 0.05.

## 3 Results

### 3.1 Baseline Characteristics

**FIGURE 1:**
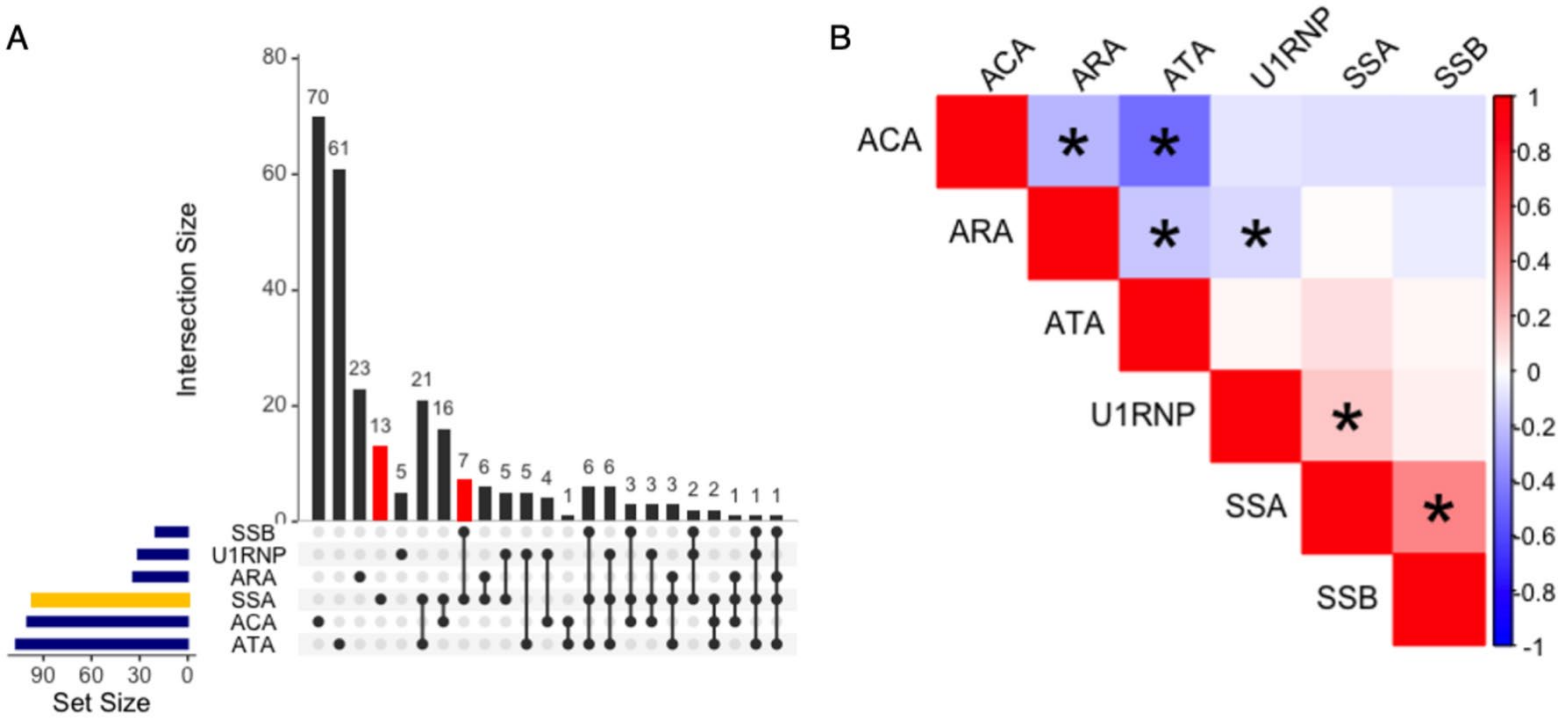
Autoantibody distribution and correlation analysis. (A) Upset plot illustrating the distribution and mutual exclusiveness of autoantibodies. ACA, anti‐centromere antibody; ARA, anti‐RNA polymerase III antibody; ATA, anti‐topoisomerase I antibody. An orange bar indicates anti‐SS‐A antibody‐positive cases, while red bars indicate isolated anti‐SS‐A antibody‐positive cases. (B) Correlation matrix illustrating mutual exclusiveness among ATA, ACA, and ARA, and statistically significant positive correlations between anti‐SS‐A and anti‐U1‐RNP seropositivity, as well as between anti‐SS‐A and anti‐SS‐B seropositivity. *p < 0.05. *p* values were calculated by Spearman's correlation test.

Table 1 summarizes the baseline characteristics of the study cohort, particularly focusing on anti‐SSA seropositivity. The total study population consisted of 307 patients, among whom 96 (31.3%) tested positive for anti‐SSA. The mean disease duration was 6.0 years with a standard deviation (SD) of 8.0 years. The follow‐up duration was significantly shorter in the anti‐SSA seropositive group compared to the anti‐SSA seronegative group (mean 5.5 years vs. 6.3 years, *p* = 0.048). Figure 1A illustrates the autoantibody distribution. While ATA, ACA, and ARA demonstrated mutual exclusivity, statistically significant positive associations were observed between anti‐SSA and anti‐U1‐RNP seropositivity, as well as between anti‐SSA and anti‐SSB seropositivity (Figure 1B). In addition, anti‐SSA and ARA were negatively correlated with statistical significance. The prevalence of dcSSc, ILD, PH, heart failure, SRC, ileus, reflux esophagitis, and myositis (PM or DM) among the whole cohort was 44.8%, 44.8%, 2.6%, 1.6%, 2.3%, 41.7%, 2.9%, and 2.3%, respectively.

**TABLE 1:** Characteristics of the subject patients at the baseline.

	*N*	Total	Anti‐SSA seropositive	Anti‐SSA seronegative	*p*
Male	307	35 (11%)	13/96 (13.5%)	22/211 (10%)	0.442
Age (years)	307	55.3 (15.5)	55 (16)	56 (15)	0.523
Disease duration (years)	307	6.0 (8.0)	5.5 (7.4)	6.3 (8.2)	0.751
Follow‐up duration (years)	307	6.6 (3.8)	5.9 (3.3)	6.8 (4.0)	0.048*
History of smoking	307	69 (22%)	20/96 (21%)	49/211 (23%)	0.768
Autoantibody					
Anti‐topo I Ab	303	107 (35%)	40/96 (42%)	67/207 (32%)	0.123
Anti‐centromere Ab	306	100 (33%)	25/96 (26%)	75/210 (36%)	0.115
Anti‐RNA polymerase III Ab	307	34 (11%)	11/96 (11%)	23/211 (11%)	0.847
Anti‐U1‐RNP Ab	306	31 (10%)	17/96 (18%)	14/210 (7%)	0.004**
Death	307	14 (5%)	7/96 (7%)	7/211 (3%)	0.143
Multi‐organ failure	14	4 (29%)			
SRC	14	3 (21%)			
ILD	14	2 (14%)			
COVID‐19	14	2 (14%)			
Pneumonia	14	2 (14%)			
Myocarditis	14	1 (7%)			
TAFRO syndrome	14	1 (7%)			
Unknown	14	2 (14%)			
Progression	307	31 (10%)	10/96	21/211 (10%)	> 0.999
Non‐progression survival, years	307	5.9 (3.7)	5.4 (3.3)	6.2 (3.9)	0.103
ILD	31	26 (84%)			
Skin sclerosis	31	5 (16%)			
Myositis	31	1 (3%)			
Arthritis	31	1 (3%)			
GI symptoms	31	1 (3%)			
Comorbidities					
PM/DM	307	7 (2%)	4/96 (4%)	3/211 (1%)	0.211
SLE	307	11 (4%)	7/96 (7%)	4/211 (2%)	0.040*
SS	307	55 (18%)	36/96 (38%)	19/211 (9%)	< 0.001***
PBC	307	10 (3%)	4/96 (4%)	6/211 (3%)	0.51
APS	307	6 (2%)	4/96 (4%)	2/211 (1%)	0.079
Raynaud's phenomenon	301	249 (83%)	80/94 (%)	169/207 (82%)	0.514
Puffy finger	222	143 (64%)	45/68 (66%)	98/154 (64%)	0.763
Nail fold bleeding	293	203 (69%)	69/92 (75%)	134/201 (67%)	0.173
Telangiectasia	235	94 (40%)	35/69 (51%)	59/166 (36%)	0.040*
Medications					
Corticosteroids	307	62 (20%)	23/96 (24%)	39/211 (19%)	0.285
Immunosuppressants	307	26 (8%)	8/96 (8%)	18/211 (9%)	> 0.999
Endothelin receptor antagonists	307	21 (7%)	7/96 (7%)	14/211 (7%)	0.811
Phosphodiesterase 5 inhibitors	307	9 (3%)	5/96 (5%)	4/211 (2%)	0.144
Beraprost	307	69 (22%)	23/96 (24%)	46/211 (22%)	0.662
Sarpogrelate hydrochloride	307	37 (12%)	9/96 (9%)	28/211 (13%)	0.45
Limaprost alfadex	307	25 (8%)	9/96 (9%)	16/211 (8%)	0.654
Angiotensin‐converting enzyme inhibitors	307	7 (2%)	1/96 (1%)	6/211 (3%)	0.441
Non‐steroidal anti‐inflammatory drugs	307	47 (15%)	14/96 (15%)	33/211 (16%)	0.866
Tocopherol nicotinate	307	88 (29%)	26/96 (27%)	62/211 (29%)	0.786
Proton pump inhibitors	307	135 (44%)	46/96 (48%)	89/211 (42%)	0.386
Laboratory tests					
White blood cells (/mm^3^)	307	6500 (2400)	6500 (2900)	6500 (2100)	0.333
Hemoglobin (g/dL)	307	12.4 (1.8)	12.5 (1.5)	12.3 (1.9)	0.404
Hematocrit (%)	307	38.4 (4.7)	38.7 (4.1)	38.3 (5.0)	0.665
Platelets (×10^4^/mm^3^)	307	25.9 (7.6)	26.0 (8.8)	25.9 (6.9)	0.618
CRP (mg/dL)	307	0.47 (1.90)	0.80 (3.10)	0.33 (0.90)	0.032*
ESR (mm/h)	300	24.6 (19.2)	30.6 (20.9)	21.8 (17.8)	0.001**
Skin involvement					
Qualitative measurement					
Diffuse cutaneous systemic sclerosis	291	130 (45%)	43/92 (47%)	87/199 (44%)	0.704
Quantitative measurement					
mRSS	270	9.9 (9.4)	9.7 (7.9)	10.0 (10.1)	0.513
Lung involvement					
Qualitative evaluation					
ILD	307	137 (45%)	60/96 (62.5%)	77/211 (36%)	< 0.001***
Quantitative evaluation					
KL‐6 (U/mL)	307	520 (497)	622 (546)	473 (468)	< 0.001***
SP‐D (ng/mL)	295	119 (108)	139 (105)	110 (109)	0.002**
%FVC (%)	303	87.0 (18.8)	83.9 (19.0)	88.5 (18.5)	0.052
%DLco (%)	297	88.1 (20.6)	84.0 (19.6)	89.9 (20.8)	0.027*
Heart involvement					
Qualitative evaluation					
Pulmonary hypertension	307	8 (3%)	3/96 (3%)	5/211 (2%)	0.709
Quantitative evaluation					
BNP (pg/mL)	296	45.6 (65.6)	50.9 (85.4)	43.1 (54.2)	0.912
LVEF (%)	292	69.1 (6.8)	68.5 (6.9)	69.4 (6.7)	0.315
*E*/*e*′	249	9.7 (3.5)	9.5 (3.6)	9.8 (3.4)	0.382
RVSP (mmHg)	285	27.1 (7.3)	27.3 (8.5)	27.0 (6.7)	0.722
Renal involvement					
Qualitative evaluation					
SRC	307	7 (2%)	1/96 (1%)	6/211 (3%)	0.441
Quantitative evaluation					
eGFR (mL/min/1.73 m^2^)	306	86.5 (27.5)	86.0 (25.6)	86.8 (28.5)	0.936
Gastrointestinal involvement					
Qualitative evaluation					
Reflux esophagitis	301	125 (42%)	46/94 (49%)	79/207 (38%)	0.101
Ileus	307	9 (3%)	3/96 (3%)	6/211 (3%)	> 0.999
Categorical evaluation					
Los Angeles classification					0.145
Grade N	278	163 (59%)	46/87 (53%)	117/191 (61%)	
Grade M	278	48 (17%)	12/87 (14%)	36/191 (19%)	
Grade A	278	46 (17%)	20/87 (23%)	26/191 (14%)	
Grade B	278	13 (5%)	5/87 (6%)	8/191 (4%)	
Grade C	278	7 (3%)	4/87 (4%)	3/191 (2%)	
Grade D	278	1 (0%)	0/87 (0%)	1/191 (1%)	
Musculoskeletal involvement					
Quantitative evaluation					
CK (U/L)	306	113 (132)	127 (172)	106 (109)	0.675

*Note:* Data are shown as *N* (%) for categorical variables and mean (standard deviation) for continuous variables.

* *p* < 0.05.

** *p* < 0.01.

*** *p* < 0.001.

### 3.2 Association Between Anti‐SSA Seropositivity and Clinical Traits

The presence of anti‐SSA was not significantly associated with age, sex, or disease duration, but significantly associated with higher presence of anti‐U1RNP seropositivity, higher inflammation markers such as CRP and ESR, higher coincidence of SLE, SS, telangiectasia, and ILD (Table 1). Worse ILD in the anti‐SSA seropositive group was indicated by barometers such as serum levels of Krebs von den Lungen 6 (KL‐6) and Surfactant protein D (SP‐D), as well as %DLco. Consistently, Spearman's correlation analysis demonstrated significant positive correlations between anti‐SSA titers and KL‐6, SP‐D, CRP, and ESR, and significant negative correlations with %FVC and %DLco (Figure S1), further supporting the association of anti‐SSA with pulmonary involvement and systemic inflammation. There was no statistically significant difference in the prevalence of dcSSc nor mRSS between the anti‐SSA seropositive and seronegative groups. Table S1 presents clinical characteristics stratified by anti‐SSA seropositivity across different autoantibody subsets. Notably, the distinctive features associated with anti‐SSA seropositivity observed in the overall population, such as elevated ESR and increased prevalence of ILD, were specifically evident only within the ATA‐positive subgroup.

### 3.3 Overall Survival Analysis

**FIGURE 2:**
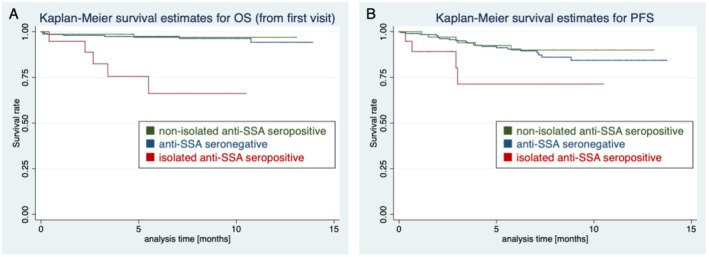
Kaplan–Meier survival curves. (A) Overall survival (OS). (B) Progression‐free survival (PFS). The *X*‐axis indicates survival time (months), and the *Y*‐axis indicates survival rate. Red lines represent patients with isolated anti‐SSA seropositivity; green lines represent patients with non‐isolated anti‐SSA seropositivity, blue lines represent anti‐SSA seronegative patients.

Table 2 presents the results of the Cox regression analysis for OS, highlighting higher age, the absence of nail fold bleeding (NFB), and the presence of SRC or ileus as significant factors in univariate analysis. Follow‐up duration was not associated with OS, indicating that this imbalance did not confound the analyses. While anti‐SSA seropositivity alone was not significantly associated with OS, isolated anti‐SSA seropositivity showed a statistically significant association with OS. Multivariate analysis incorporating these univariate factors confirmed a significant association between OS and isolated anti‐SSA seropositivity (HR: 21.7, 95% CI: 5.57–84.8; Figure 2A), along with the absence of NFB (HR: 0.12, 95% CI: 0.03–0.45) and the presence of SRC (HR: 104, 95% CI: 17.3–620). When patients were categorized into three groups—isolated anti‐SSA seropositive, non‐isolated anti‐SSA seropositive, and anti‐SSA seronegative, both the non‐isolated anti‐SSA seropositive group and the anti‐SSA seronegative group showed significantly longer OS compared with the isolated anti‐SSA seropositive group, with hazard ratios of 0.08 (95% CI: 0.02–0.43) and 0.10 (95% CI: 0.03–0.33), respectively (Figure 2A).

**TABLE 2:** Cox regression analysis of the relationship between overall survival and clinical parameters.

	Univariate analysis		Multivariate analysis	
	*N*	HR (95% CI)	*N*	HR (95% CI)
Male	302	2.28 (0.64–8.21)		
Age (years)	302	1.05* (1.01–1.09)	288	1.04 (0.99–1.09)
Disease duration (years)	302	1.03 (0.98–1.09)		
History of smoking	307	2.70 (0.94–7.80)		
Autoantibody				
ATA	298	0.46 (0.13–1.65)		
ACA	301	0.57 (0.16–2.03)		
ARA	301	1.38 (0.31–6.16)		
Anti‐U1‐RNP Ab	301	NA		
Anti‐SS‐A Ab	302	2.36 (0.83–6.77)		
Isolated anti‐SS‐A Ab	302	10.4*** (3.40–32.0)	288	21.7*** (5.57–84.8)
Raynaud's phenomenon	296	1.21 (0.27–5.43)		
Puffy finger	217	0.87 (0.22–3.50)		
Nail fold bleeding	288	0.31* (0.11–0.89)	288	0.12** (0.03–0.45)
Telangiectasia	230	0.39 (0.08–1.83)		
Medications				
Corticosteroids	302	0.33 (0.04–2.53)		
Immunosuppressants	302	NA		
Endothelin receptor antagonists	302	NA		
Phosphodiesterase 5 inhibitors	302	NA		
Beraprost	302	0.92 (0.26–3.33)		
Sarpogrelate hydrochloride	302	1.16 (0.26–5.20)		
Limaprost alfadex	302	2.28 (0.51–10.3)		
Angiotensin‐converting enzyme inhibitors	302	3.61 (0.47–27.9)		
Non‐steroidal anti‐inflammatory drugs	302	0.97 (0.22–4.32)		
Tocopherol nicotinate	302	0.40 (0.09–1.80)		
Proton pump inhibitors	302	1.94 (0.67–5.61)		
Skin involvement				
Diffuse cutaneous systemic sclerosis	287	1.43 (0.48–4.24)		
Lung involvement				
Interstitial lung disease	302	2.30 (0.77–6.88)		
Heart involvement				
Pulmonary hypertension	302	4.39 (0.56–34.0)		
Renal involvement				
SRC	302	22.0*** (6.87–70.3)	288	104*** (17.3–620)
Gastrointestinal involvement				
Reflux esophagitis	296	2.38 (0.80–7.11)		
Ileus	301	5.87* (1.31–26.3)	288	0.54 (0.10–3.01)
Musculoskeletal involvement				
Myositis	302	NA		

* *p* < 0.05.

** *p* < 0.01.

*** *p* < 0.001.

We also conducted a similar analysis by redefining OS from the first occurrence of non‐Raynaud's phenomenon—adding disease duration to the original OS—which likewise demonstrated a statistically significant association between isolated anti‐SSA seropositivity and shorter OS (HR: 25.0, 95% CI: 6.57–94.8; Table S2). When patients were categorized into three groups, redefined OS was significantly longer in both the non‐isolated anti‐SSA seropositive group and the anti‐SSA seronegative group compared with the isolated anti‐SSA seropositive group, with HRs of 0.07 (95% CI: 0.01–0.39) and 0.09 (95% CI: 0.03–0.30), respectively (Figure S2).

### 3.4 Progression‐Free Survival Analysis

Table 3 presents the Cox regression analysis results for PFS. Univariate analysis identified age, isolated anti‐SSA seropositivity, diffuse skin sclerosis, and the presence of ILD or myositis as factors associated with PFS. In multivariate analysis, isolated anti‐SSA seropositivity (HR: 4.18, 95% CI: 1.05–16.7) and the presence of diffuse skin sclerosis (HR: 2.69, 95% CI: 1.12–6.44) emerged as independent risk factors for disease progression. Follow‐up duration was not associated with PFS, indicating that follow‐up duration did not confound the analysis. When patients were categorized into three groups, PFS was also significantly longer in the non‐isolated anti‐SSA seropositive and anti‐SSA seronegative groups than in the isolated anti‐SSA seropositive group, with hazard ratios of 0.24 (95% CI: 0.07–0.88) and 0.30 (95% CI: 0.11–0.84), respectively (Figure 2B).

**TABLE 3:** Cox regression analysis of relationship between progression‐free survival and clinical parameters.

	Univariate analysis		Multivariate analysis	
	*N*	HR (95% CI)	*N*	HR (95% CI)
Male	302	1.70 (0.65–4.44)		
Age (years)	302	0.97* (0.95–0.99)	283	0.98 (0.96–1.01)
Disease duration (years)	302	0.98 (0.92–1.04)		
Follow‐up duration (years)	302	1.01 (0.99–1.02)		
History of smoking	302	0.68 (0.26–1.77)		
Autoantibody				
Anti‐topo I Ab	298	3.76** (1.77–7.99)	283	2.49 (0.86–7.22)
Anti‐centromere Ab	301	NA		
Anti‐RNA polymerase III Ab	302	0.25 (0.03–1.87)		
Anti‐U1‐RNP Ab	301	1.55 (0.60–4.05)		
Anti‐SS‐A Ab	302	1.13 (0.53–2.41)		
Isolated anti‐SS‐A Ab	302	3.31* (1.15–9.52)	283	4.18* (1.05–16.7)
Raynaud's phenomenon	296	1.37 (0.48–3.93)		
Puffy finger	217	0.90 (0.34–2.40)		
Nail fold bleeding	288	0.96 (0.44–2.10)		
Telangiectasia	230	0.35 (0.12–1.03)		
Medications				
Corticosteroids	302	1.33 (0.57–3.10)		
Immunosuppressants	302	1.12 (0.34–3.69)		
Endothelin receptor antagonists	302	1.74 (0.53–5.74)		
Phosphodiesterase 5 inhibitors	302	NA		
Beraprost	302	1.20 (0.54–2.68)		
Sarpogrelate hydrochloride	302	1.33 (0.51–3.48)		
Limaprost alfadex	302	0.45 (0.06–3.28)		
Angiotensin‐converting enzyme inhibitors	302	NA		
Non‐steroidal anti‐inflammatory drugs	302	0.63 (0.19–2.06)		
Tocopherol nicotinate	302	0.80 (0.36–1.80)		
Proton pump inhibitors	302	0.91 (0.44–1.88)		
Skin involvement				
Diffuse cutaneous systemic sclerosis	287	4.48** (1.92–10.4)	283	2.69* (1.12–6.44)
Lung involvement				
Interstitial lung disease	302	4.02** (1.79–8.99)	283	2.27 (0.86–5.98)
Heart involvement				
Pulmonary hypertension	302	NA		
Renal involvement				
SRC	302	NA		
Gastrointestinal involvement				
Reflux esophagitis	296	1.12 (0.55–2.28)		
Ileus	302	NA		
Musculoskeletal involvement				
Myositis	302	4.30* (1.01–18.2)	283	1.81 (0.38–8.63)

* *p* < 0.05.

** *p* < 0.01.

Table S3 provides further insights into ILD‐specific PFS analysis. ATA seropositivity (HR: 10.9, 95% CI: 2.48–47.8), isolated anti‐SSA seropositivity (HR: 17.0, 95% CI: 2.84–101), and diffuse skin sclerosis (HR: 3.85, 95% CI: 1.29–11.5) were independently associated with ILD‐specific PFS. When patients were categorized into three groups, ILD‐specific PFS was significantly longer in the non‐isolated anti‐SSA seropositive and anti‐SSA seronegative groups compared with the isolated anti‐SSA seropositive group, with hazard ratios of 0.20 (95% CI: 0.06–0.72) and 0.22 (95% CI: 0.08–0.62), respectively (Figure S3).

### 3.5 Characteristics of Patients With Isolated Anti‐SSA Ab Seropositivity

Table 4 outlines the characteristics of patients with isolated anti‐SSA seropositivity. Among the 20 cases, 5 patients had SS, 4 had myositis, and 1 had APS, whereas no cases of SLE or PBC were identified. Notably, some cases showed indications of autoantibodies other than anti‐SSA in antinuclear antibody testing. In fact, A‐Cube analysis detected severe disease‐associated autoantibodies such as anti‐U3‐RNP, anti‐SSSCA1, anti‐Th/To, anti‐eIF2B, and anti‐hUBF Abs in certain patients. Among the 19 cases with available A‐Cube results, all (100%) were positive for anti‐Ro52 Abs, whereas anti‐Ro60 and anti‐SSB were detected in 9 (47%) and 4 (21%) cases, respectively. Anti‐Ro60 seropositivity was observed in one of four cases with disease progression and in three of five fatal cases, while anti‐SSB seropositivity was observed in one of four progression cases and in two of five fatal cases. Nevertheless, Fisher's exact tests showed no statistically significant associations of anti‐Ro60 or anti‐SSB seropositivity with either disease progression or death. Notably, in all cases with progression (*n* = 4), treatment modifications were due to ILD. Among five deaths, two were attributed to COVID‐19 or myocarditis, while ILD, GERD, and serositis accounted for one death each. These findings indicate that high mortality associated with isolated anti‐SSA seropositivity was not driven by a single complication but rather by a heterogeneous set of fatal events, including infectious, cardiac, pulmonary, and gastrointestinal involvement.

**TABLE 4:** Clinical features of isolated anti‐SSA seropositive cases.

Case #	Age	Sex	IIF	Autoantibodies additionally revealed by A‐Cube	Comorbidities	Progression	Progressed symptom	Death	Cause of death
1	50	F	Negative	cN1A, Ro52	Myositis	+	ILD	−	
2	69	F	Speckled ×2560	Ro52	Myositis	+	ILD	−	
3	59	F	Negative	eIF2B, Ro52	SS	+	ILD	−	
4	79	F	Nucleollar ×2560	U3‐RNP, Ro52, Ro60, SSB	SS	+	ILD	−	
5	50	F	Homogenous ×40, speckled ×320	Ro52, Ro60, SSB		−		+	COVID‐19, myocarditis
6	79	F	Speckled ×2560	Ro52, Ro60	SS	−		+	Reflux esophagitis
7	53	F	Speckled ×40, nucleolar ×2560	Ro52, cN1A	SS	−		+	COVID‐19
8	56	M	Speckled ×2560	eIF2B, Ki, Ro52, Ro60, SSB	SS	−		+	Serositis, myocarditis
9	71	M	Speckled ×320, nucleolar ×2560	Th/To, Ro52		−		+	ILD
10	51	M	Speckled ×40	Ro52, Ro60		−		−	
11	83	F	Nucleolar ×2560	U3‐RNP, Ro52		−		−	
12	17	M	Speckled ×160	Ro52		−		−	
13	48	F	Speckled ×2560	Ro52, Ro60, SSB	Myositis	−		−	
14	45	F	Homogenous ×40, speckled ×40, nucleolar ×2560	CENPA, Ro52		−		−	
15	27	F	Speckled ×320	Ro52, Ro60	Myositis	−		−	
16	73	F	Speckled ×40, nucleolar ×2560	U3‐RNP, SSSCA1, DLAT, DBT, Ro52, Ro60, SSB		−		−	
17	61	F	Speckled ×40, nucleolar ×320	hUBF, Ro52, Ro60		−		−	
18	69	F	Speckled ×40	Ro52	APS	−		−	
19	59	F	Negative	Ro52		−		−	
20	74	F	Speckled ×40, nucleolar ×40	NA		−		−	

Abbreviations: APS, anti‐phospolipid antibody syndrome; IIF, indirect immunofluorescence; ILD, interstitial lung disease; NA, not available; SS, Sjogren's syndrome.

### 3.6 Sjögren's Syndrome Subgroup Analysis

Table S4 compares clinical characteristics between anti‐SSA seropositive patients with and without Sjögren's syndrome. No significant differences were identified in terms of age, sex distribution, disease duration, nor the prevalence of ILD between the two groups. However, anti‐SSA seropositive patients with SS demonstrated significantly higher seropositivity for anti‐U1RNP Abs, a higher incidence of puffy fingers, and more frequent use of tocopherol nicotinate.

## 4 Discussion

In this study of 307 Japanese patients with SSc, we identified “isolated” anti‐SSA seropositivity—defined by the absence of other major SSc‐related autoantibodies detectable through clinical laboratory testing covered by health insurance in Japan (ATA, ACA, ARA, and anti‐U1‐RNP)—as an independent predictor of both poor OS and shorter PFS (Figure 2; Tables 2 and 3). While the clinical characteristics of SSc patients with anti‐SSA seropositivity have been reported in SSc [8, 9, 10], our data suggest that the absence of concomitant SSc‐related autoantibodies may mark a unique subgroup with particularly aggressive disease trajectories from a longitudinal observation of a Japanese cohort. Consistent with previous investigations, severe organ complications—such as SRC—remained strong predictors of mortality in our cohort (Table 2). We also found the association between the absence of NFB and worse OS, which likely reflects the transition to a more advanced vasculopathic phase in SSc [22]. Additionally, we confirmed that diffuse skin sclerosis independently associates with PFS (Table 3). Moreover, when analyzing ILD‐PFS, ATA seropositivity emerged as an additional prognostic factor (Table S2). These findings are consistent with established predictors from both Western and Japanese registries [4, 23], highlighting the ongoing clinical relevance of these traditional risk factors.

Our finding that anti‐SSA seropositivity in general was not an independent predictor of poor outcomes, yet emerged as strongly prognostic when isolated, raises important considerations. First, the observed link between isolated anti‐SSA and ILD aligns with prior reports, which identified anti‐SSA seropositivity as a potential risk factor for pulmonary fibrosis in SSc [10]. Although the exact pathogenic role of anti‐SSA remains to be elucidated, our data advocate routine screening for anti‐SSA in SSc and heightened vigilance for those who harbor isolated anti‐SSA seropositivity. These patients may benefit from earlier and more frequent pulmonary imaging (e.g., high‐resolution computed tomography) and cardiopulmonary assessments, given their susceptibility to ILD. Furthermore, given the elevated inflammatory markers in this subgroup, there is a compelling rationale for investigating whether targeted immunomodulatory therapies (e.g., B‐cell depletion or cytokine blockade) might offer additional clinical benefits [24, 25]. Second, standard assays for ATA, ACA, ARA, and anti‐U1‐RNP Abs might overlook low‐titer or infrequent autoantibody specificities—such as anti‐U3‐RNP or other rare targets—that can be detected only via specialized platforms. Indeed, in a subset of “isolated” anti‐SSA‐positive patients, expanded serologic testing revealed additional autoantibodies implicated in severe SSc phenotypes. Furthermore, there might be any autoantibodies of unknown association with SSc or SS, which could be investigated by more expanded human protein arrays [26, 27]. Such hidden autoantibody repertoire could contribute to the heightened fibrotic or vasculopathic processes leading to early disease progression and increased mortality [27].

Anti‐SSA has been classically linked to SS and SLE. Nevertheless, subgroup analysis revealed no significant prognostic differences between patients with and without SS (Table S3), indicating that SS itself does not explain the elevated risk associated with isolated anti‐SSA seropositivity. Instead, anti‐SSA—particularly anti‐Ro52—may reflect distinct pathogenic mechanisms in SSc by impairing Ro52‐mediated ubiquitination activity [28], leading to increased pro‐inflammatory cytokine production, tissue injury, and progression of ILD. Although Ro52 is naturally expressed intracellularly, previous studies indicate that Ro52 can be expressed on the cell surface under specific conditions, such as oxidative stress or ultraviolet exposure [29, 30]. Recent investigations have increasingly focused on anti‐Ro52 Abs, demonstrating their independent association with ILD across various connective tissue diseases [31], their potential as biomarkers for pulmonary involvement in SSc [32], and their identification as a risk factor for PH among SSc patients [33]. In contrast, the clinical implications of anti‐Ro60 Abs in SSc remain unclear.

In our cohort, the co‐occurrence of anti‐SSA and anti‐U1‐RNP Abs was statistically significant (Figure 1B). This finding is consistent with prior reports, in which the same association has been reproducibly documented across independent cohorts from different ethnicities [8, 10], suggesting that the phenomenon is not incidental but instead reflects shared immunological mechanisms. Although these autoantibodies recognize distinct protein targets, both are directed against RNA‐binding RNP complexes. It should be noted that Ro60 (TROVE2), La (SSB), and U1‐small nuclear RNP proteins contain RNA recognition motifs (RRMs) with considerable sequence and conformational similarity, raising the possibility of cross‐reactive immune responses. Supporting this concept, Routsias et al. demonstrated that immunization of rabbits with the RRM of La/SSB not only elicited anti‐SSB responses but also induced anti‐U1‐RNP Abs [34], providing experimental evidence for intermolecular and even interparticle epitope spreading within related RNP families.

Several limitations warrant consideration. This was a single‐center, retrospective study, potentially introducing selection bias and limiting generalizability to other ethnic groups. Although we employed comprehensive clinical evaluations, the intensity and frequency of follow‐up could vary, potentially affecting the timing of disease progression endpoints. In addition, determination of the direct cause of death in SSc is inherently challenging; fatal events in our cohort were heterogeneous and sometimes combined. The small number of deaths further reduced statistical power, particularly for cause‐specific analyses. Consequently, the contribution of isolated anti‐SSA seropositivity to cause‐specific mortality remains uncertain and warrants confirmation in larger, longitudinal cohorts. Moreover, our expanded autoantibody testing was restricted to cases positive for isolated anti‐SSA; thus, we cannot rule out the possibility that certain autoantibody subpopulations might be underrepresented in other subgroups. Furthermore, differentiation between anti‐Ro52 and anti‐Ro60 Abs was similarly limited to these specific cases. Prospective multicenter studies with expanded autoantibody panels are needed to confirm our findings, explore the functional role of rarer autoantibody specificities, and elucidate the difference between anti‐Ro52 and anti‐Ro60 seropositivity. From a viewpoint of therapeutic intervention, mechanistic investigations into how anti‐SSA or autoreactive B‐cells targeting SS‐A antigen drive tissue injury in systemic sclerosis could uncover novel therapeutic targets, especially with the recent emergence of selective therapies aimed at autoreactive B‐cells, such as chimeric autoantibody receptor T‐cells [35, 36, 37].

In summary, our findings highlight isolated anti‐SSA seropositivity as an important, independent marker of poor prognosis in Japanese patients with SSc. These results emphasize the need for expanded serologic profiling and vigilant clinical monitoring in this unique subgroup, which appears especially prone to aggressive organ involvement and early disease progression. Future prospective studies should clarify whether therapeutic interventions tailored to high‐risk autoimmune profiles can improve outcomes in patients who present with isolated anti‐SSA seropositivity.

## Ethics Statement

The whole study was approved by the ethical committee of The University of Tokyo Hospital (approval number: 0695 and 2023051G).

## Consent

Written informed consent was obtained from the participants.

## Conflicts of Interest

K.M. Matsuda received a lecture fee from Fushimi Pharmaceutical Co. Ltd. The other authors declare no conflicts of interest.

## Supporting information


**Figure S1:** Correlation between serum levels anti‐SSA antibodies and clinical parameters. *ρ*: Spearman's rho. Bed line and shaded area represent the regression line and its 95% confidence interval.

**Figure S2:** Kaplan–Meier survival curves for overall survival framed from the first occurrence of non‐Raynaud's phenomenon. The *X*‐axis indicates survival time (months), and the *Y*‐axis indicates survival rate. Red line represents patients with isolated anti‐SSA seropositivity; green line represents patients with non‐isolated anti‐SSA seropositivity, blue line represents anti‐SSA seronegative patients.

**Figure S3:** Kaplan–Meier survival curves for interstitial lung disease‐specific progression‐free survival. The *X*‐axis indicates survival time (months), and the *Y*‐axis indicates survival rate. Red line represents patients with isolated anti‐SSA seropositivity; green line represents patients with non‐isolated anti‐SSA seropositivity, blue line represents anti‐SSA seronegative patients.

**Table S1:** Characteristics of the subject patients at the baseline by disease‐specific autoantibody profiles.

**Table S2:** Cox regression analysis of relationship between overall survival from disease onset and clinical parameters.

**Table S3:** Cox regression analysis of relationship between interstitial lung disease progression‐free survival and clinical parameters.

**Table S4:** Characteristics of the subject patients with anti‐SSA seropositivity by concurrence of Sjogren's syndrome.

## Data Availability

The data that support the findings of this study are available on request from the corresponding author. The data are not publicly available due to privacy or ethical restrictions.
