# Supplementary figures and images for: Isolated Anti‐SS‐A Antibody Seropositivity as a Poor Prognostic Factor in Systemic Sclerosis: Insights From a Cohort of 307 Cases
Source: J Dermatol. 2025 Nov 22;53(1):41–53. doi: 10.1111/1346-8138.70055 (PMC12784790; doi:10.1111/1346-8138.70055)

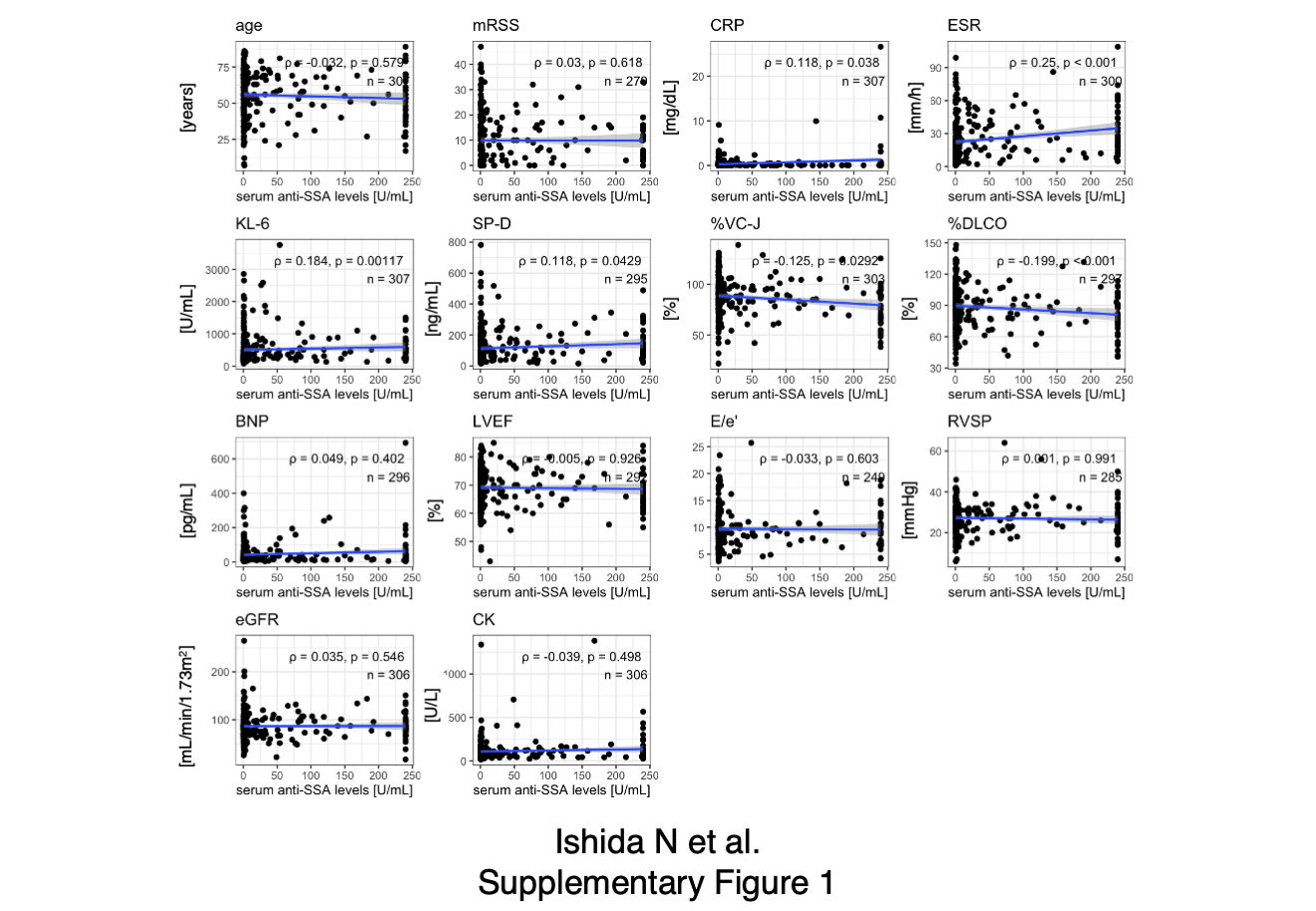

Supplement: Supplementary file 1 — Figure S1: Correlation between serum levels anti‐SSA antibodies and clinical parameters. ρ: Spearman's rho. Bed line and shaded area represent the regression line and its 95% confidence interval. [file JDE-53-41-s007.tiff]

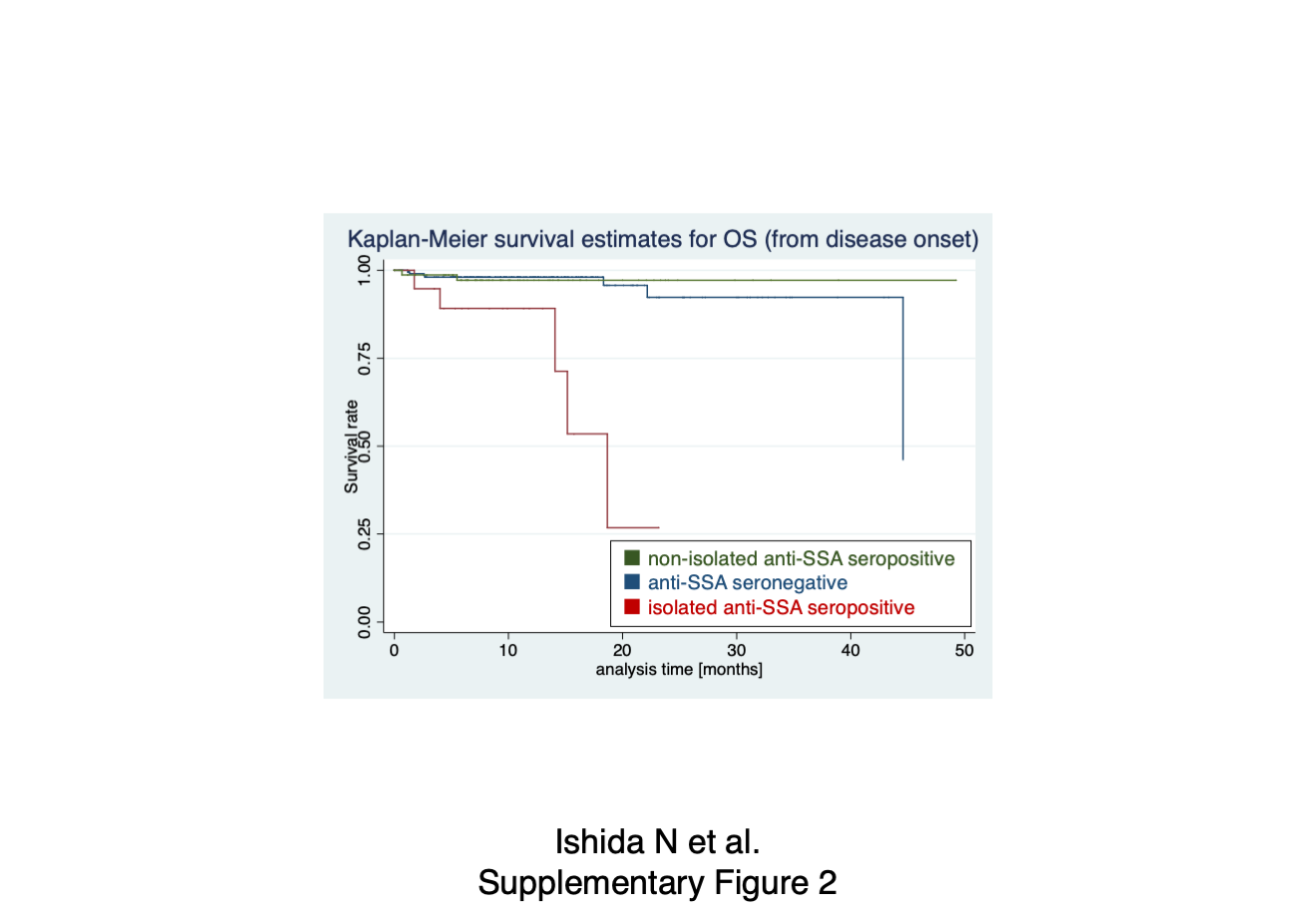

Supplement: Supplementary file 2 — Figure S2: Kaplan–Meier survival curves for overall survival framed from the first occurrence of non‐Raynaud's phenomenon. The X‐axis indicates survival time (months), and the Y‐axis indicates survival rate. Red line represents patients with isolated anti‐SSA seropositivity; green line represents patients with non‐isolated anti‐SSA seropositivity, blue line represents anti‐SSA seronegative patients. [file JDE-53-41-s004.tiff]

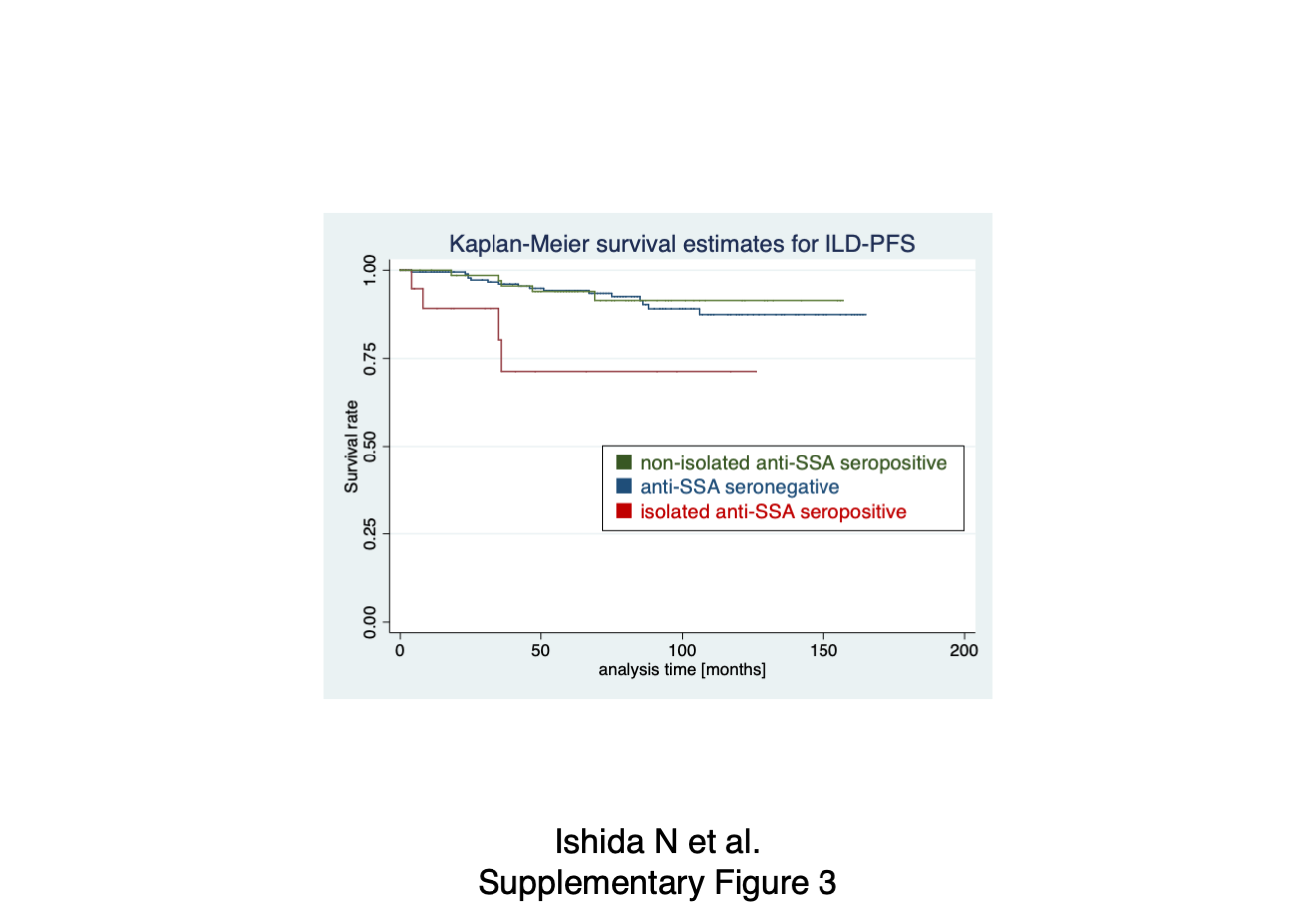

Supplement: Supplementary file 3 — Figure S3: Kaplan–Meier survival curves for interstitial lung disease‐specific progression‐free survival. The X‐axis indicates survival time (months), and the Y‐axis indicates survival rate. Red line represents patients with isolated anti‐SSA seropositivity; green line represents patients with non‐isolated anti‐SSA seropositivity, blue line represents anti‐SSA seronegative patients. [file JDE-53-41-s006.tiff]
